# Supplementary material for: A developmental increase of inhibition promotes the emergence of hippocampal ripples
Source: Nat Commun. 2024 Jan 25;15:738. doi: 10.1038/s41467-024-44983-z (PMC10810866; doi:10.1038/s41467-024-44983-z)
Supplement: Supplementary file 1 — Supplementary Information [file 41467_2024_44983_MOESM1_ESM.pdf]

Supplementary Materials for

A developmental increase of inhibition promotes the emergence of hippocampal ripples

Irina Pochinok[1], Tristan M. Stöber[2], Jochen Triesch[2], Mattia Chini[1]*§ & Ileana L. Hanganu-Opatz[1]*§

[1]Institute of Developmental Neurophysiology, Center for Molecular Neurobiology (ZMNH), Hamburg Center of Neuroscience (HCNS), University Medical Center Hamburg-Eppendorf, 20251 Hamburg, Germany.

[2]Frankfurt Institute for Advanced Studies, 60438 Frankfurt am Main, Germany

* Corresponding authors: Mattia Chini
mattia.chini@zmnh.uni-hamburg.de
Falkenried 94, 20251 Hamburg, Germany
https://orcid.org/0000-0002-5782-9720

Ileana L. Hanganu-Opatz
hangop@zmnh.uni-hamburg.de
Falkenried 94,20251 Hamburg, Germany
https://orcid.org/0000-0002-4787-1765

§ Equal contribution

Supplementary materials include:

Supplementary Figures S1 to S9
Supplementary Tables S1 to S5

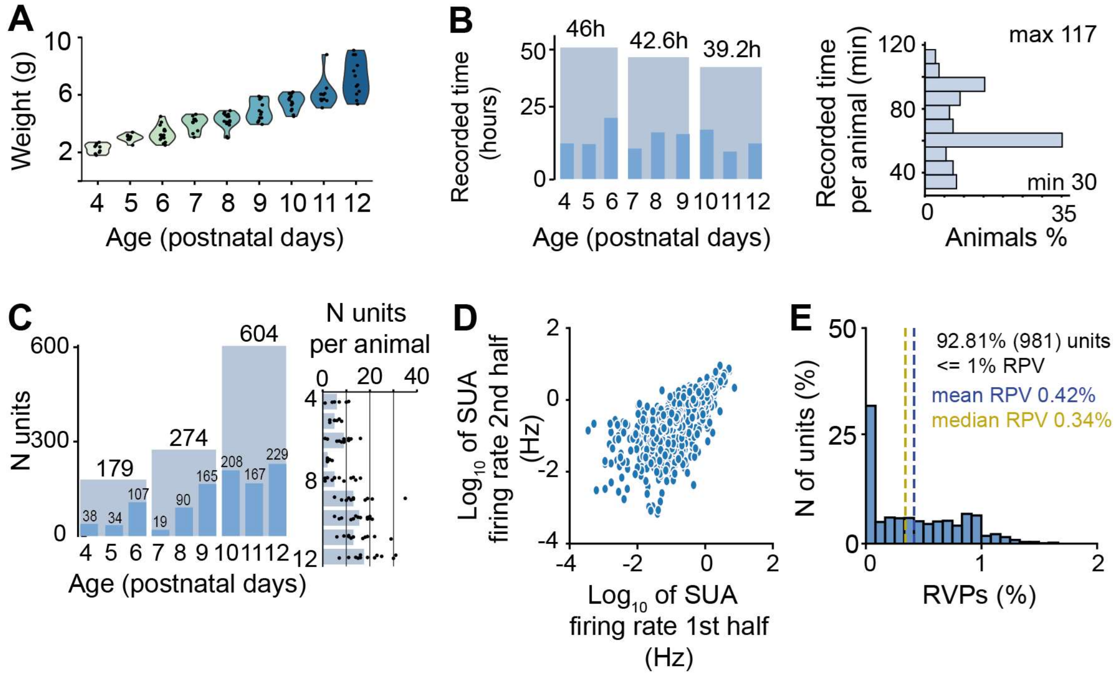

Supplementary Fig. 1. Features of the experimental dataset.

(A) Violin plot displaying the weight of P4-P12 mice (n=111 mice).

(B) Distribution of the duration lengths over age (left) and mice (right) (n=111 mice).

(C)-(E) SUA features.

(C) Distribution of unit numbers over age (left) and mice (right). On the right plot, each dot corresponds to an individual animal, and the bar plot indicates the median number of units.

(D) Correlation of firing rates during the 1st vs the 2nd half of the recording. Pearson coefficient 0.81, $p < 0.0001$, Spearman coefficients 0.76, $p < 0.0001$, two-sided.

(E) Percentage of refractory period violations.

In **(A)** dots correspond to individual animals. In **(D)** dots correspond to individual single units. In **(A)** the shaded area represents the probability distribution density of the variable. Source data are provided as a Source Data file.

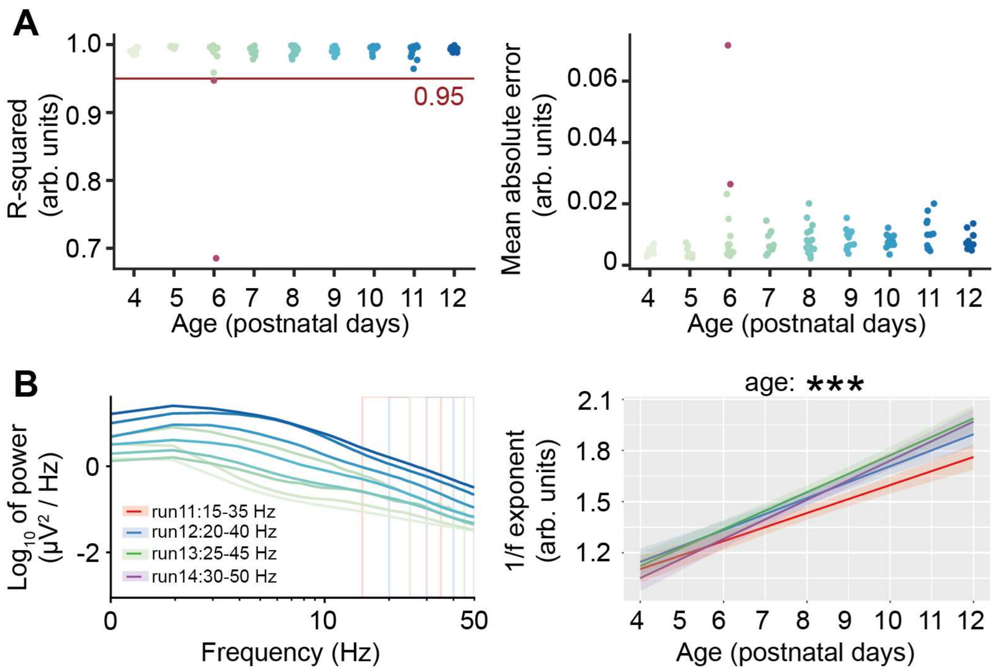

Supplementary Fig. 2. Quality metrics for the power spectra parametrization procedure (FOOOF) in the low-frequency band.

(A) R-squared (left) and mean absolute error (right) of the power spectra parametrization in the 25-45 Hz frequency range as shown in Figure 1E. Two red dots correspond to the two mice with low-quality parametrization that were removed from the dataset.

(B) Power spectral density of P4-P12 mice with four highlighted frequency bands used for power spectra parametrization (left), and corresponding 1/f exponent over age for the four different frequency bands (right). Color codes for the different frequency bands. Linear mixed-effects model with two-way interaction (fit frequency range x age), mouse as random effect, effect of age, $p < 0.0001$, two-sided, Tukey correction for multiple comparisons.

In **(A)** dots correspond to individual animals. In **(B)**, asterisks indicate a significant effect of age. *** $p < 0.001$. Source data are provided as a Source Data file.

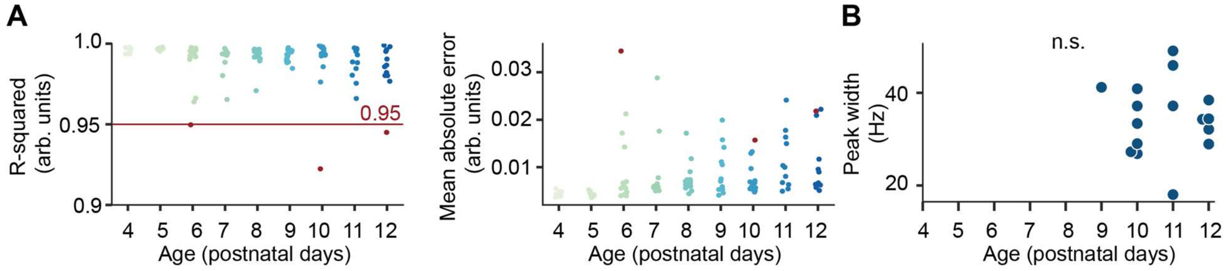

Supplementary Fig. 3. Quality metrics for the power spectra parametrization procedure (FOOOF) in the fast frequency band.

(A) R-squared (left) and mean absolute error (right) of the power spectra parametrization in 70-200 Hz frequency range as shown in Figure 3E-G. Three red dots correspond to the three mice with low-quality parametrization that were removed from the dataset.

(B) Scatter plot displaying the width of the detected fast frequency peaks of P4-P12 mice. Linear model, 95% CI [-4.88; 4.20], p = 0.88, two-sided.

In **(A)** and **(B)**, dots correspond to individual animals. Source data are provided as a Source Data file.

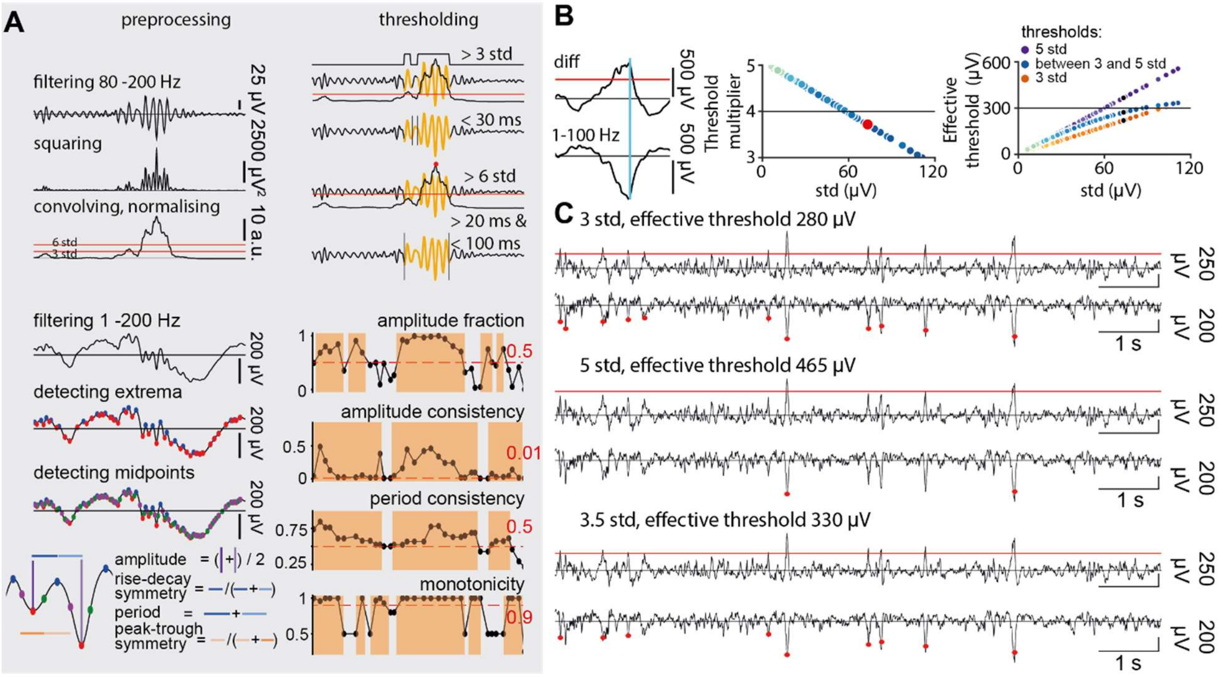

Supplementary Fig. 4. Schematic of the SPW-R detection approach.

(A) Schematic of the two ripple detection methods applied to 300 ms long LFP traces recorded in the stratum pyramidale as shown in Figure 4A (right).

(B) Schematic of the sharp wave detection method applied to 300 ms long LFP trace recorded in the stratum radiale as shown in Figure 4A (right). Left, signal obtained by subtracting the signal recorded 100 µm below from the signal recorded 100 µm above the channel in CA1 str. pyramidale (top) and LFP trace from str. radiale with one SPW (bottom). The red line indicates the threshold applied for SPW detection. Middle, scatter plot displaying the threshold multiplier (in standard deviations) as a function of the LFP standard deviation. Right, scatter plot displaying the threshold in µV as a function of the LFP standard deviation for 5 std threshold, 3 std threshold and adaptive threshold spaced between 3 and 5 std.

(C) A characteristic example of discrepancies in sharp wave detection outcomes resulting from different threshold selection. Source data are provided as a Source Data file.

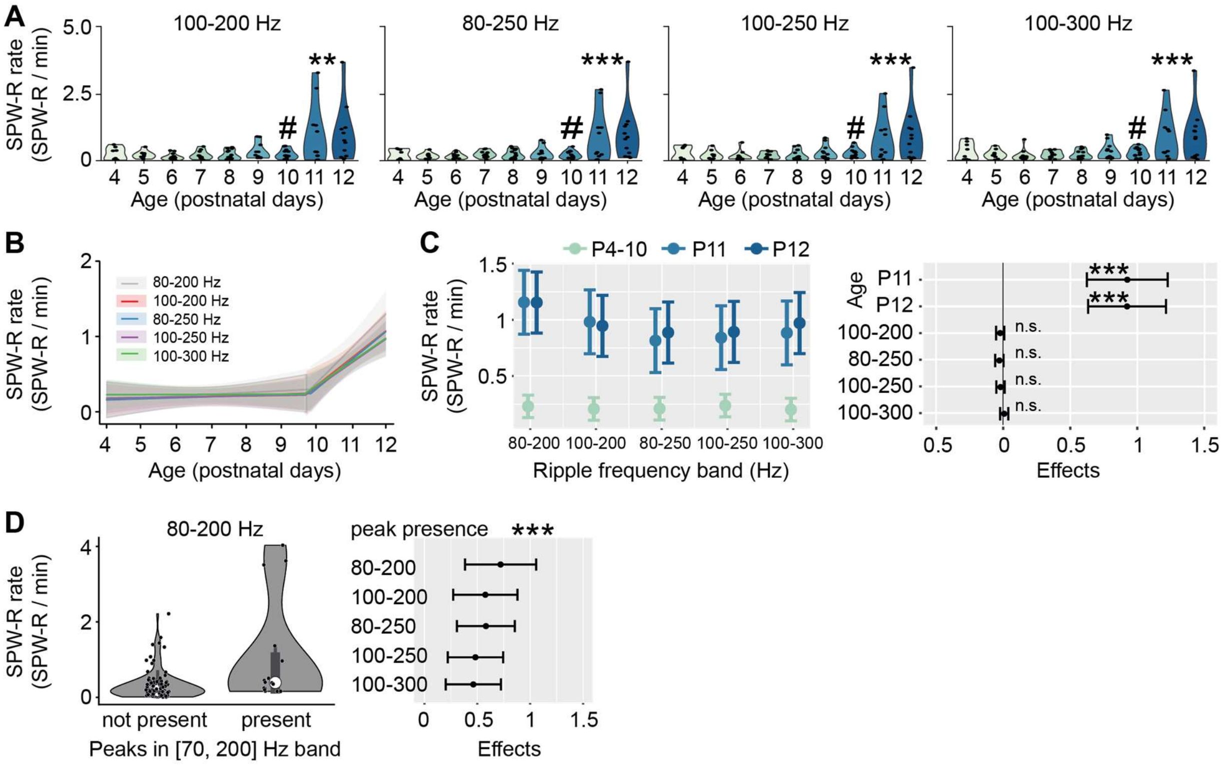

Supplementary Fig. 5. Quality metrics for the SPW-R detection approach.

(A) Violin plot displaying the SPW-R rate of P4-P12 mice (n=111 mice) in four frequency bands. From left to right: 100-200 Hz, 80–250 Hz, 100–250 Hz, 100-300 Hz). Linear model with segmented fit, two-sided, detailed statistical results are provided in Supplementary Table S5.

(B) Line plot displaying predicted values of SPW-R rate of P4-P12 mice (n=111 mice) in five frequency bands. Color codes for the different frequency bands. Shaded area represents 95% confidence interval.

(C) Predicted values of SPW-R rate for P4-P10, P11 and P12 mice (n=111 mice) in five frequency bands (left) and an effect of age and frequency band on SPW-R rate (right). Linear mixed-effects model with two-way interaction (ripple frequency band x age), mouse as random effect, two-sided, Tukey correction for multiple comparisons, detailed statistical results are provided in Supplementary Table S5.

(D) Violin plot with a box plot displaying SPW-R rate of P4-P12 mice (n=108 mice) with absent (92 mice) and present (16 mice) high frequency power spectra peaks (left), and an effect of peak on SPW-R rate in five frequency bands (right). Linear model, $p < 10^{-4}$ (peak effect, 80-200Hz), p = 0.00029 (peak effect, 100-200Hz), p = 0.00056 (peak effect, 100-300Hz), p = < 10^{-4} (peak effect, 80-250Hz), p = 0.000402 (peak effect, 100-250Hz), two-sided.

In **(A)** and **(D)**, the shaded area represents the probability distribution density of the variable and dots correspond to individual animals. In **(D)**, data in the box plot are presented as median (central white circle), interquartile range (thick line) and whiskers (thin lines) extending to the

maxima/minima at most 1.5 times the interquartile range. In **(A)** and **(C)** asterisks indicate a significant effect of age. In **(D)**, asterisks indicate a significant effect of a presence of high frequency oscillatory peak in power spectra. ** $p < 0.01$, *** $p < 0.001$. Hash (#) indicates estimated breakpoints in linear piece-wise regression. Source data are provided as a Source Data file.

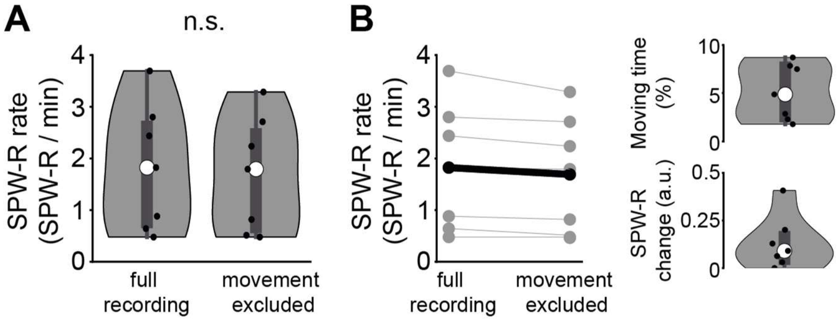

Supplementary Fig. 6. Effect of animal movements on SPW-R rate in the hippocampal CA1 area of P11-P12 mice.

(A) Violin plot with a box plot displaying the SPW-R rate of P11-P12 mice (n=7 mice) calculated on full recorded period versus calculated only on periods with no movements. Linear model 95% CI [-1.49; 1.23], $p = 0.837$, two-sided.

(B) Scatter plot displaying the SPW-R rate of P11-P12 mice (n=7 mice) calculated on full recorded period and periods with no movements (left) and violin plot with a box plot displaying the percentage of movement time (right, top) and change in SPW-R rate between full recorded period and periods with no movements (right, bottom).

In **(A)** and **(B, right)**, data in the box plot are presented as median (central white circle), interquartile range (thick line) and whiskers (thin lines) extending to the maxima/minima at most 1.5 times the interquartile range. The shaded area on the violin plot represents the probability density distribution of the variable. In **(B)**, gray dots on the scatterplot correspond to individual animals while black dots represent mean values. Source data are provided as a Source Data file.

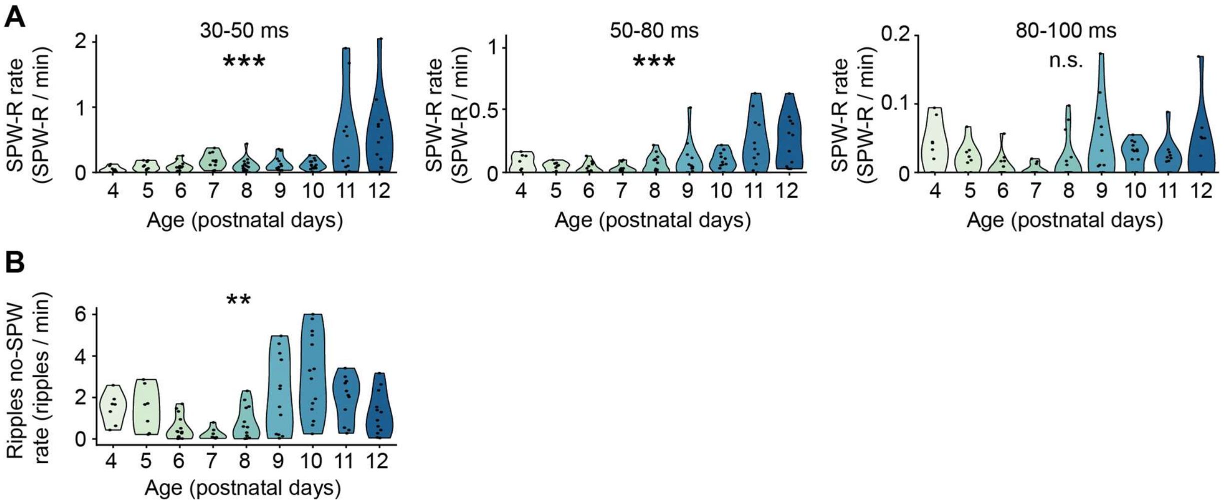

Supplementary Fig. 7. Developmental changes in SPW-R rate and ripples-without SPW rate in the hippocampal CA1 area of P4-P12 mice.

(A) Violin plot displaying the SPW-R rate of P4-P12 mice (n=111 mice) for ripples of varying duration. Left to right: 30-50 ms, 50-80 ms, 80-100 ms. Linear model, 30-50ms: 95% CI [0.04; 0.08], p = $p < 10^{-5}$; 50-80ms: 95% CI [0.02; 0.04], $p < 10^{-6}$; 80-100ms: 95% CI [-0.001; 0.004], p = 0.2348; two-sided.

(B) Violin plot displaying the rate of ripples without sharp waves of P4-P12 mice (n=111 mice). Linear model 95% CI [0.073; 0.293], p = 0.00129, two-sided.

In **(A)** and **(B)**, the shaded area represents the probability distribution density of the variable and dots correspond to individual animals. In **(A)** and **(B)** asterisks indicate a significant effect of age. ** $p < 0.01$, *** $p < 0.001$. Source data are provided as a Source Data file.

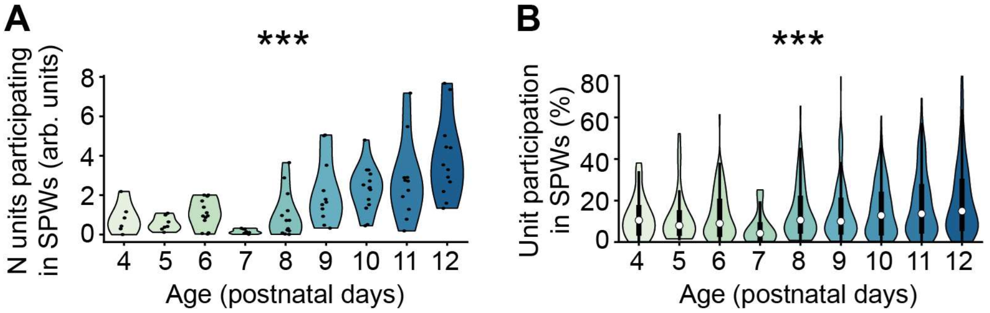

Supplementary Fig. 8. Single unit participation in SPWs/SPW-Rs in the hippocampal CA1 area of P4-P12 mice.

(A) Violin plot displaying the number of units simultaneously contributing to SPWs/SPW-Rs events of P4-P12 mice (n=111 mice). Linear mixed-effects model, mouse as random effect, 95% CI [0.30; 0.53], $p < 10^{-9}$, two-sided.

(B) Violin plot with a box plot displaying single units' participation in SPWs/SPW-Rs of P4-P12 mice (n=111 mice). Linear mixed-effects model, mouse as random effect, 95% CI [0.60; 1.66], $p < 10^{-4}$, two-sided.

In **(A)** and **(B)**, the shaded area represents the probability distribution density of the variable. In **(A)**, dots correspond to individual animals. In **(B)**, data in the box plot are presented as median (central white circle), interquartile range (thick line) and whiskers (thin lines) extending to the maxima/minima at most 1.5 times the interquartile range. In **(A)** and **(B)**, asterisks indicate a significant effect of age. *** $p < 0.001$. Source data are provided as a Source Data file.

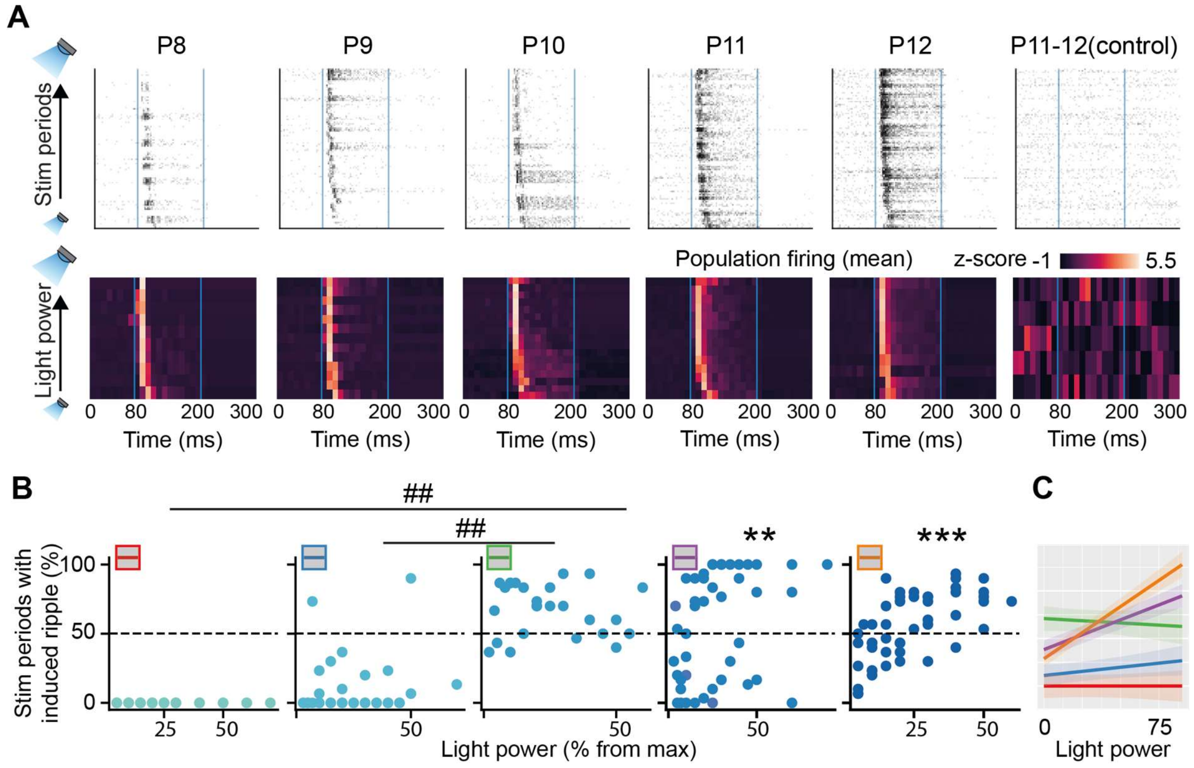

Supplementary Fig. 9. Effect of light power intensity on the evoked LFP and SUA activity of P8-P12 mice.

(A) Raster plot (top) and heatmap (bottom) displaying the population firing in response to optogenetic stimulation of increasing light intensity.

(B) Percentage of stimulation periods with iHFO. Blue dots correspond to the response induced by a specific light power. Linear mixed-effects model with two-way interaction (light power x age), mouse as random effect, two-sided, Tukey correction for multiple comparisons, detailed statistical results are provided in Supplementary Table S5.

(C) A linear mixed-effect model prediction trained on data shown in **(B)**. Each line corresponds to the light power-response rate relationship for a particular age.

In **(B)**, an asterisk indicates a significant effect of light power. ** $p < 0.01$, *** $p < 0.001$. Hash (##) indicates a significant difference in the effect of age. ## $p < 0.01$. Source data are provided as a Source Data file.

Supplementary Table 1. Spiking neural network model description.

Model summary	
Populations	Two: excitatory(E, $N_E = 12000$) and inhibitory(I, $N_I = 200$)
Topology	—
Connectivity	Random connections
Neuron model	Leaky integrate-and-fire
Synapse model	Biexponential conductance-based
Plasticity	—
Input	Time-dependent conductances with a Gaussian profile, delivered to a subset of E.
Measurements	Spike activity, membrane potential

Connectivity				
**Name**	**Source**	**Target**	**Connection probability**	**Conduction delay**
EE	E	E	$p_{EE} = 0.0164$	1ms
IE	E	I	$p_{IE} = 0.1$	1ms
EI	I	E	$p_{EI} = 0.1$	1ms
II	I	I	$p_{II} = 0.2$	1ms

Neuron model	
Subthreshold dynamics	$C_P \frac{dV(t)}{dt} = I^P_{leak} + I^P_{exc} + I^P_{inh} + I^P_{noise}, P \in \{E, I\}$<br><br>$I^P_{leak} = g^P_{leak}(E^P_{rest} - V(t))$<br>$I^P_{exc} = (g^P_{exc}(t) + k g_{ext}(t))(E^P_{exc} - V(t))$<br>$I^P_{inh} = g^P_{inh}(t)(E^P_{inh} - V(t))$<br>$I^P_{noise} = \sigma \sqrt{\frac{2 g^P_{leak}}{C_P}} \xi$<br>$\sigma$ is the standard deviation of the intrinsic membrane noise, ξ is the Ornstein-Uhlenbeck process which drives the noise<br><br>$k = \begin{cases} 1 & \text{if } P = E \\ 0 & \text{if } P = I \end{cases}$
Spiking	Every time a membrane potential reaches the threshold V^P_{thr}, a spike is emitted, V is set to V^P_{reset} and clamped for a refractory period τ^P_{ref}

Synapse model	
Dynamics	$\frac{dg^E_\alpha(t)}{dt} = \frac{\left(\frac{\tau^P_{\alpha,r}}{\tau^P_{\alpha,d}}\right)^{\frac{\tau^P_{\alpha,d}}{\tau^P_{\alpha,r} - \tau^P_{\alpha,d}}} s^P_\alpha(t) - g^P_\alpha(t)}{\tau^P_{\alpha,d}}$<br>$\frac{ds^P_\alpha(t)}{dt} = -\frac{s^P_\alpha(t)}{\tau^P_{\alpha,r}}$<br><br>$P \in \{E, I\}, \alpha \in \{exc, inh\}$

Supplementary Table 2. Neuronal parameters.

Parameter	E cells	I cells	Definition
C_P	$C_E = 275\ pF$	$C_I = 100\ pF$	membrane capacitance
E^P_{rest}	$E^E_{rest} = -67\ mV$	$E^I_{rest} = -65\ mV$	resting potential
E^P_{inh}	$E^E_{inh} = -68\ mV$	$E^I_{inh} = -75\ mV$	inhibitory reversal potential
E^P_{exc}	$E^E_{exc} = 0\ mV$	$E^I_{exc} = 0\ mV$	excitatory reversal potential
g^P_{leak}	$g^E_{leak} = 25\ nS$	$g^I_{leak} = 10\ nS$	leak conductance
V^P_{thr}	$V^E_{thr} = -50\ mV$	$V^I_{thr} = -52\ mV$	firing threshold
τ^P_{ref}	$\tau^E_{ref} = 2\ ms$	$\tau^I_{ref} = 1\ ms$	refractory period
V^P_{reset}	$V^E_{reset} = -67\ mV$	$V^I_{reset} = -65\ mV$	reset potential

Supplementary Table 3. Synaptic parameters.

Parameter	Synapses on E cells	Synapses on I cells	Definition
$g^P_{exc,peak}$	$g^E_{exc,peak} = 0.9\ nS$	$g^I_{exc,peak} = 3.0\ nS$	peak conductance,AMPA
$g^P_{inh,peak}$	$g^E_{inh,peak} = 9.0\ nS$	$g^I_{inh,peak} = 5.0\ nS$	peak conductance,GABA
$\tau^P_{inh,d}$	$\tau^E_{inh,d} = 2.0\ ms$	$\tau^I_{inh,d} = 1.2\ ms$	decay time constant, GABA
$\tau^P_{inh,r}$	$\tau^E_{inh,r} = 0.4\ ms$	$\tau^I_{inh,r} = 0.45\ ms$	rise time constant,GABA
$\tau^P_{exc,d}$	$\tau^E_{exc,d} = 1.8\ ms$	$\tau^I_{exc,d} = 1.2\ ms$	decay time constant, AMPA
$\tau^P_{exc,r}$	$\tau^E_{exc,r} = 0.5\ ms$	$\tau^I_{exc,r} = 0.5\ ms$	rise time constant,AMPA

Supplementary Table 4. Simulated developmental changes in inhibition.

increase in perisomatic inhibition (EI connections)	30 configurations<br>$c\_gei \times g^{E}_{inh,peak}$<br>$c\_gii \times g^{I}_{inh,peak}$<br>$c\_gii = 1$<br>$c\_gei \in \{0.095 + 0.0005k \vert k \in \{0, 1, ..., 10\}\}$<br>$c\_gei \in \{0.1 + 0.1k \vert k \in \{1, 2, ..., 19\}\}$
increase in reciprocal inhibition in I population (II connections)	35 configurations<br>$c\_gei \times g^{E}_{inh,peak}$<br>$c\_gii \times g^{I}_{inh,peak}$<br>$c\_gei = 1$<br>$c\_gii \in \{0.05 + 0.01k \vert k \in \{0, 1, ..., 4\}\}$<br>$c\_gii \in \{0.095 + 0.0005k \vert k \in \{1, 2..., 10\}\}$<br>$c\_gii \in \{0.1 + 0.1k \vert k \in \{1, 2..., 19\}\}$

Supplementary Table 5. Detailed statistical results.

Figure	Metric	Factor	n	Statistical method	Test value	p-values
Figure 1						
Fig 1C	Percentage of time in active periods	Age	110 mice, 1 P12 mouse excluded (outlier)	Generalized linear model, binomial family, logit link function, R glm function, family='binomial'	46.94	p<0.0001, two-sided
Fig 1D	SUA firing rate, log10	Age	95 mice, 1057 single units	Generalized linear mixed-effects model, gamma family, log link function, random effect mouse, R glmer function, family=Gamma(link='log')	9.43	p<0.0001, two-sided
Fig 1F	1/f exponent	Age	109 mice, 2 P6 mice excluded (FOOOF fit R-squared < 0.95)	Linear model with segmented fit, R lm and segmented functions	7.12, -1.49	p<0.0001 (age slope P4-P10), p=0.14 (age slope P10-P12), two-sided
Figure 3						
Fig 3B	PSD in [80-200] Hz, log10	Age	111 mice	Generalized linear model, gamma family, log link function, R glm function, family=Gamma(link='log')	9.29	p<0.0001, two-sided

Fig 3E	PSD offset	Age	108 mice, 3 mice excluded (1 P6, 1 P10, 1 P12, FOOOF fit R-squared < 0.95)	Generalized linear model, gamma family, log link function, R glm function, family=Gamma(link='log')	8.57	p<0.0001, two-sided
Fig 3G left	PSD peak power, log10	Age	16 peaks from 16 mice P9-P12	Linear model, R lm function	1.90	p=0.08, two-sided
Fig 3G right	PSD peak central frequency	Age	16 peaks from 16 mice P9-P12	Linear model, R lm function	0.61	p=0.55, two-sided
Figure 4						
Fig 4C	SPW rate	Age	111 mice	Linear model with segmented fit, R lm and segmented functions	4.42, -4.79, 3.91	p<0.0001 (age slope P4-P7), p<0.0001 (age slope P7-P10), p=0.00017(age slope P10-P12), two-sided
Fig 4D	SPW-R rate	Age	111 mice	Linear model with segmented fit, R lm and segmented functions	-0.03, 3.41	p=0.97 (age slope P4-P10), p=0.00096 (age slope P10-P12), two-sided
Fig 4E	Percentage of SPWs with ripples	Age	111 mice	Linear model with segmented fit, R lm and segmented functions	-0.72, 3.00	p=0.47 (age slope P4-P9), p=0.0034 (age slope P9-P12), two-sided
Fig 4F	Ripple length	Condition (age group: P4-P9, P10-P11)	6312 ripples from 111 mice	Linear mixed-effects model, random effect mouse, R lmer and emmeans functions	7.65	p<0.0001, two-sided, post hoc analysis with Tukey multiple comparison corrections (emmeans)

Fig 4G	Number of short and long ripples	Age	6312 ripples from 111 mice	Linear model, R lm function	-0.533, 4.012	p=0.6108 (short), p=0.00511 (long), two-sided
Fig 4H	Percentage of short and long ripples	Age	6312 ripples from 111 mice	Linear model, R lm function	-3.842, 3.842	p=0.006355 (short), p=0.00636 (long), two-sided
Figure 5						
Fig 5B	Population firing rate	Age	95 mice	Linear model, R lm function	3.31, 6.20	p=0.0014(SPW-R, 5B left), p<0.0001(SPW no R, 5B right), two-sided
	Population firing rate	Condition (presence or absence of a ripple)	95 mice	Linear mixed-effects model, random effect mouse, R lmer function	7.98	p<0.0001, two-sided
Fig 5D	Unit spikes per SPW (%)	Age	95 mice	Linear model, R lm function	-3.09, 3.489, 5.053	1 spike: p=0.0176<br>2-3 spikes: p=0.01014<br>>=4 spikes: p= 0.00148, two-sided
Figure 6						
Fig 6A	mCherry+ cells per mm^2	Age	22 mice	Linear model, R lm function	-1.15	p=0.261, two-sided
Fig 6D	Percentage of stim periods with ripples	Age	22 mice	Generalized linear model, binomial family, logit link function, R glm function, family='binomial'	20.56	p<0.0001, two-sided

Fig 6E	Unit spikes per sweep (%)	Age	22 mice	Linear model, R lm function	-10.24, 10.242, 5.338	1 spike: p=0.00198 2-3 spikes: p=0.00198 >=4 spikes: p=0.0129, two-sided
Figure 7						
Fig 7C	Modulation index SPW-R rate	Condition (control or cre+)	15 mice (6 control, 9 cre+)	Linear model, R lm and emmeans functions	-2.74	p=0.017, two-sided, post hoc analysis with Tukey multiple comparison corrections (emmeans)
Fig 7F	Modulation index firing rate	Condition (control or cre+)	15 mice (6 control, 9 cre+), 126 single units (53 control, 73 cre+)	Linear mixed-effects model, random effect mouse, R lmer and emmeans functions	-2.25	p=0.042, two-sided, post hoc analysis with Tukey multiple comparison corrections (emmeans)
Figure 8						
Fig 8B	Population firing rate	Age	85 mice	Linear model, R lm function	1.62, 0.11	P=0.108(all SPW, 8B left) p=0.909(SPW-R, 8B right), two-sided
	Population firing rate	Condition (presence or absence of a ripple)	85 mice	Linear mixed-effects model, random effect mouse, R lmer function	4.407	p<0.0001, two-sided
Figure S1						
Fig S1D	SUA firing rate, log10	Condition (recording half)	95 mice, 1057 single units	Pearson correlation Spearman correlation, scipy.stats.pearsonr and	0.81, 0.76	p<0.0001, p<0.0001, two-sided

				scipy.stats. spearmanr		
Figure S2						
Fig S2B right	1/f exponent	Age	109 mice, 2 P6 mice excluded (FOOOF fit R-squared < 0.95)	Linear mixed-effects model with two-way interaction (FOOOF fit range * age), random effect mouse, R lmer, emtrends and emmeans functions	5.06	p<0.0001, two-sided, post hoc analysis with Tukey multiple comparison corrections (emmeans, emtrends)
Figure S3						
Fig S3B	PSD peak width	Age	16 peaks from 16 mice P9-P12	Linear model, R lm function	-0.16	p=0.87, two-sided
Figure S5						
Fig S5A 100-200Hz	SPW-R rate	Age	111 mice	Linear model with segmented fit, R lm and segmented functions	0.399, 3.229	p=0.691 (age slope P4-P10), p=0.00165 (age slope P10-P12) , two-sided
Fig S5A 80-250Hz	SPW-R rate	Age	111 mice	Linear model with segmented fit, R lm and segmented functions	0.435, 3.824	p=0.664 (age slope P4-P10), p=0.000221 (age slope P10-P12) , two-sided
Fig S5A 100-250Hz	SPW-R rate	Age	111 mice	Linear model with segmented fit, R lm and segmented functions	0.227, 3.396	p=0.820 (age slope P4-P10), p=0.00096 (age slope P10-P12) , two-sided
Fig S5A 100-300Hz	SPW-R rate	Age	111 mice	Linear model with segmented fit, R lm and segmented functions	0.037, 3.388	p=0.970 (age slope P4-P10), p=0.000986 (age slope P10-P12) , two-sided

Fig S5C	SPW-R rate	Age, Frequency band	111 mice	Linear mixed-effect model with two-way interaction (ripple frequency band * age), random effect mouse, R lmer and emmeans function	6.025, 6.249, -1.451, 0.448, -1.839, -1.304	$p < 10^{-7}$ (age effect, P11), $p < 10^{-8}$ (age effect, P12), p = 0.147 (frequency band effect, 100-200Hz), p = 0.655 (frequency band effect, 100-300Hz), p = 0.067 (frequency band effect, 80-250Hz), p = 0.193 (frequency band effect, 100-250Hz), two-sided, post hoc analysis with Tukey multiple comparison corrections (emmeans)
Fig S5D	SPW-R rate	Condition (high frequency peak presence)	108 mice	Linear model, R lm function	4.246, 3.757, 3.562, 4.237, 3.659	$p < 10^{-4}$ (peak effect, 80-200Hz), p = 0.00029 (peak effect, 100-200Hz), p = 0.00056 (peak effect, 100-300Hz), p = $< 10^{-4}$ (peak effect, 80-250Hz), p = 0.000402 (peak effect, 100-250Hz), two-sided
Figure S6						
Fig S6A	SPW-R rate	Condition	7 mice	Linear model, R lm function	-0.210	p = 0.837, two-sided

		(recording type)				
Figure S7						
Fig S7A 30-50 ms	SPW-R rate	Age	111 mice	Linear model, R lm function	4.915	$p < 10^{-5}$, two-sided
Fig S7A 50-80 ms	SPW-R rate	Age	111 mice	Linear model, R lm function	5.356	$p < 10^{-6}$, two-sided
Fig S7A 80-100 ms	SPW-R rate	Age	111 mice	Linear model, R lm function	1.195	p = 0.2348, two-sided
Fig S7B	R rate	Age	111 mice	Linear model, R lm function	3.305	p = 0.00129, two-sided
Figure S8						
Fig S8A	N units per SPW	Age	95 mice	Linear mixed-effects model, random effect mouse, R lmer function	6.996	$p < 10^{-9}$, two-sided
Fig S8B	Unit participation in SPWs (% of SPWs)	Age	95 mice	Linear mixed-effects model, random effect mouse, R lmer function	4.196	$p < 10^{-4}$, two-sided
Figure S9						
Fig S6B	Percentage of stim periods with ripples	Age	22 mice	Linear mixed-effects model with two-way interaction (Light power * age), random effect mouse, R lmer, emtrends and emmeans functions	na<br>0.661<br>-0.316<br>2.788<br>4.444	p=0.999 (light power slope at P8),<br>p=0.509 (light power slope at P9),<br>p=0.753 (light power slope at P10),

						p=0.006 (light power slope at P11),<br>p<0.0001 (light power slope at P12),<br>two-sided,<br>post hoc analysis with Tukey multiple comparison corrections (emmeans, emtrends)
