# Supplementary material for: A developmental increase of inhibition promotes the emergence of hippocampal ripples
Source: Nat Commun. 2024 Jan 25;15:738. doi: 10.1038/s41467-024-44983-z (PMC10810866; doi:10.1038/s41467-024-44983-z)
Supplement: Supplementary file 3 — Reporting Summary [file 41467_2024_44983_MOESM3_ESM.pdf]

nature portfolio

Corresponding author(s): Ileana Hanganu-Opatz
Mattia Chini

Last updated by author(s): Dec 22, 2023

Reporting Summary

Nature Portfolio wishes to improve the reproducibility of the work that we publish. This form provides structure for consistency and transparency in reporting. For further information on Nature Portfolio policies, see our Editorial Policies and the Editorial Policy Checklist.

Statistics

For all statistical analyses, confirm that the following items are present in the figure legend, table legend, main text, or Methods section.

n/a	Confirmed	
☐	☒	The exact sample size (n) for each experimental group/condition, given as a discrete number and unit of measurement
☐	☒	A statement on whether measurements were taken from distinct samples or whether the same sample was measured repeatedly
☐	☒	The statistical test(s) used AND whether they are one- or two-sided<br>*Only common tests should be described solely by name; describe more complex techniques in the Methods section.*
☐	☒	A description of all covariates tested
☐	☒	A description of any assumptions or corrections, such as tests of normality and adjustment for multiple comparisons
☐	☒	A full description of the statistical parameters including central tendency (e.g. means) or other basic estimates (e.g. regression coefficient) AND variation (e.g. standard deviation) or associated estimates of uncertainty (e.g. confidence intervals)
☐	☒	For null hypothesis testing, the test statistic (e.g. F, t, r) with confidence intervals, effect sizes, degrees of freedom and P value noted<br>*Give P values as exact values whenever suitable.*
☒	☐	For Bayesian analysis, information on the choice of priors and Markov chain Monte Carlo settings
☒	☐	For hierarchical and complex designs, identification of the appropriate level for tests and full reporting of outcomes
☐	☒	Estimates of effect sizes (e.g. Cohen's d, Pearson's r), indicating how they were calculated

Our web collection on statistics for biologists contains articles on many of the points above.

Software and code

Policy information about availability of computer code

Data collection	In vivo electrophysiological data were collected using Digital Lynx SX multichannel extracellular amplifier (Neuralynx) with the Cheetah acquisition software (v.6, Neuralynx). Brain slices for cell quantification were imaged using a binocular microscope with a resolution of 5,760 × 3,600 pixels. Image acquisition was performed using a 405 nm laser for DAPI staining and a 568 nm laser for mCherry labeling. Video tracking was performed using camera UI-3360CP-NIR-GL R2 (iDS imaging, Germany) and uEye Cockpit software (iDS imaging).
Data analysis	Custom scripts written in MATLAB (R2019a, MathWorks, USA), Python (v.3.8.3) and R (v.4.1.3). Python packages: FOOOF v.1.0.0, bycycle v.1.0.0, brainrender v.2.0.5.0, neo v.0.9.0.dev, klusta v.3.0.16, phy v.2.01b1. Image analysis and cells quantification: FIJI Image J v.2.0, Cellpose v.2.2.2. Spiking neural network modeling: brian2 v.2.4.2, python v.3.9.6. Video analysis: DeepLabCut v.2.3.6.<br>Custom code repositories: https://github.com/iinnpp/spwr_snn (DOI: 10.5281/zenodo.10424461), https://github.com/OpatzLab/HanganuOpatzToolbox.

For manuscripts utilizing custom algorithms or software that are central to the research but not yet described in published literature, software must be made available to editors and reviewers. We strongly encourage code deposition in a community repository (e.g. GitHub). See the Nature Portfolio guidelines for submitting code & software for further information.

Data

Policy information about availability of data

All manuscripts must include a data availability statement. This statement should provide the following information, where applicable:

- Accession codes, unique identifiers, or web links for publicly available datasets
- A description of any restrictions on data availability
- For clinical datasets or third party data, please ensure that the statement adheres to our policy

Full spontaneous activity dataset (Local field potentials (LFP) and single unit activity (SUA)), optogenetic dataset, chemogenetic dataset and movement control dataset are available at the open source GIN repository https://gin.g-node.org/iinnpp/ripples_emergence_inhibition (DOI: 10.12751/g-node.bcds1m).

Research involving human participants, their data, or biological material

Policy information about studies with human participants or human data. See also policy information about sex, gender (identity/presentation), and sexual orientation and race, ethnicity and racism.

Reporting on sex and gender	N/A
Reporting on race, ethnicity, or other socially relevant groupings	N/A
Population characteristics	N/A
Recruitment	N/A
Ethics oversight	N/A

Note that full information on the approval of the study protocol must also be provided in the manuscript.

Field-specific reporting

Please select the one below that is the best fit for your research. If you are not sure, read the appropriate sections before making your selection.

☒ Life sciences ☐ Behavioural & social sciences ☐ Ecological, evolutionary & environmental sciences

For a reference copy of the document with all sections, see nature.com/documents/nr-reporting-summary-flat.pdf

Life sciences study design

All studies must disclose on these points even when the disclosure is negative.

Sample size	Sample sizes were determined based on similar experiments carried out by the group of the supervising authors (Chini, M., Pfeffer, T., & Hanganu-Opatz, I. (2022). An increase of inhibition drives the developmental decorrelation of neural activity. eLife, 11. https://doi.org/10.7554/eLife.78811; Ahlbeck, J., Song, L., Chini, M., Bitzenhofer, S. H., & Hanganu-Opatz, I. L. (2018). Glutamatergic drive along the septo-temporal axis of hippocampus boosts prelimbic oscillations in the neonatal mouse. eLife, 7. https://doi.org/10.7554/eLife.33158; Kostka, J. K., & Hanganu-Opatz, I. L. (2023). Olfactory-driven beta band entrainment of limbic circuitry during neonatal development. The Journal of Physiology. https://doi.org/10.1113/JP284401).
Data exclusions	Datapoints were excluded from the analysis on the following figures: Fig. 1C (1 P12 mouse, exclusion criteria - outlier based on 1.5IQR rule), Fig1. E and F (2 P6 mice excluded, exclusion criteria - FOOOF fit R-squared < 0.95), Fig. 3E (3 mice excluded, 1 P6, 1 P10, 1 P12, exclusion, criteria - FOOOF fit R-squared < 0.95), Fig. S2B (2 P6 mice excluded, exclusion criteria - FOOOF fit R-squared < 0.95)
Replication	In this study, we used datasets independently collected by two people. The reported effects were consistent and comparable across both sets of data. In the paper, results based on pooled dataset are reported. In the Materials and Methods section, we provided exact descriptions of methods used for data analysis and experimental procedures.<br>The dataset is uploaded to the open-access GIN repository (https://gin.g-node.org/iinnpp/ripples_emergence_inhibition, DOI: 10.12751/g-node.bcds1m) and the source code is available on github (code for analysis of ephys data https://github.com/OpatzLab/HanganuOpatzToolbox, code for spiking neural network https://github.com/iinnpp/spwr_snn).
Randomization	Fig.1, Fig.3, Fig.4, Fig.5, Fig.8A,B, Fig. S1-S8: Mice were allocated to specific groups based on age. The age was determined based on the day of the vaginal plug detection (embryonic day 0.5) and the day of the birth (postnatal day 0).<br>Fig. 6, Fig. 8C-E, Fig. S9: Mice were allocated to specific groups based on plasmid used during in utero electroporation (with or without channelrhodopsin-2).<br>Fig. 7. Mice were allocated to specific groups based on their genotype or presence or absence of virus expression.
Blinding	Fig.1, Fig.3, Fig.4, Fig.5, Fig.8A,B, Fig. S1-S8: The investigators were not blinded to group allocation (age groups) during the data collection because during the investigated time period (first two postnatal weeks) mice change their appearance and grow in size. The investigator

performing the experiment can therefore visually determine the age of the mouse. During the data analysis, the investigators were blinded to the age group allocation. All collected data (LFP and SUA) were pulled in one dataset not including age information and analyzed together. The age information was aligned with data/analysis results for the visualization and statistical analysis (evaluating the effect of age).
Fig. 6, Fig. 8C-E, Fig. S9 and Fig. 7: The investigators were blinded to group allocation during data collection and analysis because the presence/absence of virus expression and/or genotype were not known at the time point of electrophysiological recording and analyzing the data. The group information was aligned with data/analysis results for the visualization and statistical analysis.

Reporting for specific materials, systems and methods

We require information from authors about some types of materials, experimental systems and methods used in many studies. Here, indicate whether each material, system or method listed is relevant to your study. If you are not sure if a list item applies to your research, read the appropriate section before selecting a response.

Materials & experimental systems

n/a	Involved in the study
☒	☐ Antibodies
☒	☐ Eukaryotic cell lines
☒	☐ Palaeontology and archaeology
☐	☒ Animals and other organisms
☒	☐ Clinical data
☒	☐ Dual use research of concern
☒	☐ Plants

Methods

n/a	Involved in the study
☒	☐ ChIP-seq
☒	☐ Flow cytometry
☒	☐ MRI-based neuroimaging

Animals and other research organisms

Policy information about studies involving animals; ARRIVE guidelines recommended for reporting animal research, and Sex and Gender in Research

Laboratory animals	Experiments were performed on C57Bl/6J and Dlx5/6-Cre (Tg(dlx5a-cre)1Mekk/J, Jackson Laboratory) mice of both sexes, at the age of P4-12. Timed-pregnant mice were housed either individually or in groups of two at a 12 hr light/12 hr dark cycle with ad libitum access to water and food at a room temperature of 21°C and humidity at 43%. The day of vaginal plug detection was defined as embryonic day (E) 0.5, while the day of birth was considered postnatal day (P) 0.
Wild animals	The study did not involve wild animals.
Reporting on sex	Experiments were performed on mice of both sexes and sex was assigned by visual inspection. Information about sex was included into the dataset and sex was considered in study design. Both sexes were pooled together because no significant effect of sex on studied parameters was detected.
Field-collected samples	The study did not involve field-collected samples.
Ethics oversight	All experiments were approved by the University Medical Center Hamburg-Eppendorf guidelines and institutional animal welfare officer. All procedures were performed in compliance with German Animal Welfare Act and were approved by the State Authority of Hamburg (Behörde für Justiz und Verbraucherschutz, Amt für Verbraucherschutz, Lebensmittelsicherheit und Veterinärwesen), Germany (N18/015, N19/121).<br>In accordance with the 3 Rs guidelines for the use of animals in research, part of the data has been previously acquired in the lab and newly analyzed for the purposes of the present study.

Note that full information on the approval of the study protocol must also be provided in the manuscript.
